# Craniofacial Sleep Medicine: The Important Role of Dental Providers in Detecting and Treating Sleep Disordered Breathing in Children

**DOI:** 10.3390/children9071057

**Published:** 2022-07-15

**Authors:** Tammarie Heit, Bea Janine Tablizo, Martina Salud, Fan Mo, Mandip Kang, Mary Anne Tablizo, Manisha Witmans

**Affiliations:** 1 Avalon Dental, Edmonton, AB T6B 3T7, Canada; drtheit@gmail.com; 2 Department of Pediatrics, Philippine General Hospital, Taft Avenue, Manila 1000, Philippines; beatablizo@gmail.com; 3 Ateneo School of Medicine and Public Health, Pasig 1604, Philippines; martisalud220@gmail.com; 4 Department of Internal Medicine, University of California San Francisco-Fresno, Fresno, CA 93701, USA; fan.mo@ucsf.edu (F.M.); mbhkang10@gmail.com (M.K.); mtablizomd@gmail.com (M.A.T.); 5 Department of Pediatrics, Stanford University School of Medicine, Palo Alto, CA 94304, USA; 6 Division of Pulmonary and Sleep Medicine, Valley Children’s Hospital, Madera, CA 93636, USA; 7 Department of Pediatrics, Faculty of Medicine & Dentistry, University of Alberta, Edmonton, AB T6G 2B7, Canada

**Keywords:** craniofacial, sleep medicine, dentistry, pediatrics, OSA, polysomnography

## Abstract

Obstructive sleep apnea (OSA) is a clinical disorder within the spectrum of sleep-related breathing disorders (SRDB) which is used to describe abnormal breathing during sleep resulting in gas exchange abnormalities and/or sleep disruption. OSA is a highly prevalent disorder with associated sequelae across multiple physical domains, overlapping with other chronic diseases, affecting development in children as well as increased health care utilization. More precise and personalized approaches are required to treat the complex constellation of symptoms with its associated comorbidities since not all children are cured by surgery (removal of the adenoids and tonsils). Given that dentists manage the teeth throughout the lifespan and have an important understanding of the anatomy and physiology involved with the airway from a dental perspective, it seems reasonable that better understanding and management from their field will give the opportunity to provide better integrated and optimized outcomes for children affected by OSA. With the emergence of therapies such as mandibular advancement devices and maxillary expansion, etc., dentists can be involved in providing care for OSA along with sleep medicine doctors. Furthermore, the evolving role of myofunctional therapy may also be indicated as adjunctive therapy in the management of children with OSA. The objective of this article is to discuss the important role of dentists and the collaborative approach between dentists, allied dental professionals such as myofunctional therapists, and sleep medicine specialists for identifying and managing children with OSA. Prevention and anticipatory guidance will also be addressed.

## 1. Introduction

Sleep-related breathing disorders (SRDB) involve a clinical spectrum of respiratory sleep disorders, which can include chronic snoring, upper airway resistance syndrome (UARS), and obstructive sleep apnea syndrome (OSAS). These terminologies are collectively used to describe abnormal breathing during sleep that can result in gas exchange abnormalities and/or sleep disruption. The spectrum of disorders ranges in presentation, pathophysiology, severity, and associated sequelae based on age. Obstructive sleep apnea (OSA) is characterized by repeated episodes of partial or complete upper airway collapsibility during sleep, with subsequent repetitive arousals which can result in gas exchange abnormalities (oxygen desaturations and elevated carbon dioxide). It is estimated that 1–4% of children suffer from pediatric OSA [1]. Untreated OSA in children is associated with neurobehavioral and cognitive sequelae as well as long-term consequences of metabolic, endocrine, and cardiovascular disease [2,3]. Bhattacharjee et al. showed in their retrospective review across various academic centers that only 27.2% had complete resolution of OSA post adenotonsillectomy using AHI as the primary determinant. They reported that residual disease (defined as abnormal overnight polysomnograms) is common in older children, those with severe OSA pre surgery, and children with asthma. [4]. What was not addressed in that review was the role of family history, craniofacial profile, or malocclusion identified in dentistry which also could be some other reasons for persistent disease. More precise and personalized, targeted approaches are required to treat the complex constellation of symptoms with their associated comorbidities since not all children are cured by surgery (removal of tonsils and adenoids). As treatment strategies are developing for the spectrum of SRBD, including OSA, dentists and medical professionals can work together to optimize airway functioning and overall health in children to prevent or alleviate morbidity and mortality resulting from untreated SBRD. Dental sleep medicine has already emerged and is involved in treating adults. Recently, more attention is devoted to addressing OSA in children.

Prevention, early recognition, and reversal of suboptimal development of the mandible and maxilla and compensatory dental function leading to disease may be a very important paradigm shift compared to treating disease. Dentists can help with their knowledge of jaw and facial development to intercede and optimize craniofacial development to change the phenotype and promote healing and health without medication or surgery [5]. Cohorts involving children are starting to appear in the literature as dentists use their knowledge from adults to help children [6]. The objective of this article is to discuss the collaborative approach between dentists and sleep medicine specialists for managing children with OSA to optimize airway function and breathing for restorative sleep.

## 2. Risk Factors

Adenotonsillar hypertrophy is one of the main risk factors for developing OSA in children, and its prevalence reaches a peak between the ages of two and eight years when the lymphoid tissue is disproportionately large in relation to the craniofacial profile [2,7]. Obesity is also an important risk factor for OSA in children and adults. Mechanisms of the contribution of obesity are related to the presence of fat at the level of the pharynx as well as abdominal obesity, which decreases respiratory function [8]. Each increment in BMI above the 50th percentile is associated with a 10% increase in risk for OSA [7]. However, not all obese children have or develop OSA, which implies that there may be other factors also involved in mitigating the risk for OSA.

Other risk factors that have been reported include lingual tonsil hypertrophy, and prematurity [3]. Ethnicity also seems to play a role, as does lower socioeconomic status. Inflammation involving the airway from allergic rhinitis [7,9] or a history of upper and lower respiratory tract infections as well as environmental tobacco smoke have also been associated with OSA [7]. Many genetic syndromes with associated craniofacial abnormalities are also at higher risk for OSA and include Trisomy 21, Pierre Robin sequence, Prader Willi syndrome, achondroplasia, Apert syndrome, and Crouzon syndrome [3]. SRBD can occur in any age group, from cradle to grave, and therefore warrants routine screening and evaluation.

Risk factors for OSA from a dental perspective may involve the craniofacial profile. Attention to the underlying craniofacial profile and optimizing the structure and function may play a role in not only preventing lifelong morbidity and mortality associated with OSA but potentially prevention of the clinical disorder in the first place. One study by Guillemineault linked apparent life-threatening events, OSA, and facial dysmorphia and reported that as early as six months, those with sleep apnea were already presenting with mild facial dysmorphia [10]. Some of the children with abnormal breathing early in life went on to develop OSA by five years of age [10]. Malocclusion has been associated with OSA but is not causally proven.

Dental-medical collaboration is key and oral health care providers can be a first line screening resource for disease risk as an important component of an integrated public health initiative [11]. Greenberg and Glick identified in 2012 that ‘screening and monitoring for systemic disease risk in a dental setting were valuable components toward more effective disease prevention, control, and health care delivery’. In a national survey, dentists felt it was important to screen for medical conditions and were willing to refer to physicians for follow-up care [12]. This is an ideal situation in the detection, treatment, and prevention of OSA since dentists are also part of the treatment solution. 

## 3. Pathophysiology

The pathophysiological factors that contribute to developing OSA are related to the anatomy, which may result in a reduction of airway caliber and factors that promote airway collapsibility (intrinsic and extrinsic factors) [13]. The pediatric airway has been observed to correlate with the Starling resistor model and P crit with the degree of upper airway obstruction [13]. Detailed discussions about the pathophysiology of OSA are discussed in another paper in the pediatric OSA issue.

Emerging evidence suggests that the craniofacial profile also plays a role in the pathophysiology of OSA since the airway caliber is influenced by the surrounding structures. Upper air dimensions and craniofacial morphology are closely related. Studies have shown that a certain subset of children have certain craniofacial features that are linked to OSA. The dento-craniofacial characteristics in non-syndromic children that may be associated with increased risk for sleep disordered breathing are anterior open bite, large overjet due to mandibular retrognathia, cross bite, and narrow and high arch palate. The development of the jaws and dental occlusion may be affected by a narrow upper airway. Decreased palatal width may be a risk factor due to decreased upper airway size [14]. 

It makes sense that the form and function of the upper airway and craniofacial are interrelated and influence each other. For example, abnormal form (such as a tongue tie) leads to altered compensatory function (affected sucking and swallowing), which leads to abnormal form (enlarged posterior tongue muscles affecting the shape and size of the palate) [15]. In addition, it is widely believed that chronic nasal obstruction from chronic allergies, enlarged adenoids, or turbinate leads to mouth breathing and possibly narrowed upper palate which contribute to OSA. There may also be a role in the interaction of genes and the environment as our eating habits have changed so dramatically since the invention of bottled formula delivered through artificial nipples and soft, processed foods [16]. Although these factors have not been systematically studied in medicine, these theories are well understood in the dental field and the need for cross collaboration is becoming more obvious.

## 4. Clinical Features

Clinical features of OSA can vary greatly based on age group, as can the clinical presentation. In general, clues to SRBD, including OSA, tend to affect five main domains: sleep duration and sleep quality, breathing effort, feeding ability, growth, and development. Most of the concerns are often reported by parents given that the children themselves either are sleeping or only recognize and report the complaints and symptoms at an older age when the impairment affects daytime function. Infants with more severe symptoms tend to present with noisy breathing, apparent life-threatening events [17], and/or failure to thrive. Delayed development (speech, milestones, etc.) may be recognized as a later sign. Snoring and labored breathing or increased work of breathing are the most reported symptoms in young children. The definition and frequency of snoring vary in the literature for determining the presence of OSA. Frequent habitual snoring, defined as the presence of loud snoring at least three nights/week, is reported [7]. However, in the context of low tone, or neuromuscular weakness, snoring may not be present or obvious. Other reported symptoms can include nocturnal sweating, paradoxical breathing, mouth breathing, nocturnal enuresis, and restlessness with frequent position changes. In addition, clinicians should also focus on any signs of attention deficit and hyperactivity or any behavioral problems as these daytime symptoms are commonly present among pediatric populations, especially in the context of fragmented or non-restorative nighttime sleep [3]. Checking the weight is important as obesity has been an identified risk factor. Night terrors, either related to developmental age or a function of sleep fragmentation, may also be seen in toddlers and preschoolers [13]. Depending on the severity, there can be associated hypoxemia and/or hypoventilation with sleep disruption and/or fragmentation which cannot be determined by clinical evaluation alone. Objective measurement of gas exchange by monitoring breathing during sleep, ideally a sleep study is critical to establish the diagnosis and document severity as the clinical assessment is insufficient.

### Intraoral and Extraoral Examination Findings

Despite the prevalence of OSA in the pediatric population [1], there is limited literature on the topic of findings during dental examination suggestive of OSA in children. Clues that suggest either orthopedic or craniofacial features are associated with OSA can be quite variable. It is imperative to have a high index of suspicion to facilitate timely detection and diagnosis of OSA. It stands to reason that altered airway mechanics or airway obstruction would affect the craniofacial growth by altering forces and resulting in postural skeletal abnormalities and soft tissue changes [13]. 

The phenotype, generally speaking, of an optimal normal airway from an oral perspective, is to have a maxilla that is big enough to accommodate 32 fully erupted teeth that are straight and in class one occlusion by the time they are adolescent [18]. Ideally, the child will be breathing through the nose with the tongue sitting up against the upper palate. Mouth breathing that is both visible and audible is considered pathologic [19]. Nasal breathing with lips lightly resting together is normal and should be silent indicative of non-resistant airflow. The professional should not be able to see (motion of soft tissues or exudates) or hear their patient breathe through the nose. The maxilla and the supporting teeth of the maxilla are a foundational part of the upper jaw, the landing pad for the teeth of the mandible, and the top block of the craniofacial skeleton. This helps to support the posture of the body of the face, thereby affecting the optimal function of the adherent soft tissues. Anything deviating from that is considered compensatory and suboptimal [20]. The evidence found in the resulting malocclusion makes the individual subject to reduced function, further compensation by the body posture, and altered biofeedback mechanisms that can increase predisposition to OSA [6,19].

Hyponasal speech, chronic mouth breathing and open mouth posture with compensatory craniofacial developmental changes should be recognized by clinicians and dentists as it is a sign of nasal obstruction and adenoidal hypertrophy. The specific cephalometric stereotypic features that are noted in those with OSA from adenoidal hypertrophy include a long face. The adenoidal facies are associated with labial incompetence and an increased mandibular plane angle. Speech delay related to eustachian tube dysfunction or recurrent ear infections can also be associated with adenoidal hypertrophy. The most common features assessed during the oral examination of a child with OSA include hypertrophy of the tonsils. Tonsillar hypertrophy is associated with mouth breathing, a posterior-rotated mandible creating a mandibular discrepancy to the cranial base which diminishes the physical airway behind it. Compensation is seen in the forward posture of the head. Other features may include an elongated soft palate, a high arched palate, or a longer or larger tongue [21,22]. Enlarged tongue muscles (and obstructive) may be seen resulting from tongue tie or from compensatory work demanded of it in response to the posturing of the jaw (aberrant tongue posture). This affects optimal function as demonstrated by the release of the tongue tie to improve breastfeeding in infants [23,24,25,26,27,28] and myofunctional therapy improving OSA in children and adults [29,30]. The tongue tie has been shown to be a root cause of maxillary deficiency and linked to obstructive sleep apnea in children based on the work of Dr. Christian Guilleminault [15]. The presence of mouth breathing may also negatively affect the development of craniofacial features. As Ngiam et al. and Lee et al. have demonstrated, features that develop from mouth breathing include a long face, lower frontal facial height, and a reduced inter-maxillary space [21,22]. A reduced inter-maxillary space will reduce space for the tongue in which the tongue will be displaced posteriorly into the retroglossal airway space. An adapted tongue posture may exhibit as low and protruded forward. The non-mouth breathing individuals with OSA have a deep bite with the type II skeletal class and mandibular retraction. There is a high and retracted position of the tongue. Consistently reported findings in the literature (Rossi et al.) include decreased mandibular and maxillary lengths and its retrusion, increased total anterior facial height, a larger craniocervical angle, decreased posterior airway space, and an inferiorly positioned hyoid bone [31].

The Mallampati score is a score based on anatomical structures that are visualized by opening the mouth and protrusion of the tongue [32]. It can be used as a predictor of OSA in older school-age children but its value in infants and young children is not known. A higher score is linked to a higher likelihood of sleep apnea in older children and adults [33].

In a recent study, Lee et al. revealed that preschool children with mild OSA are more likely to present with features such as retrognathic mandible and increased overjet [34]. Crooked teeth, crowding, and tongue tie can indicate a small maxilla, narrow palate, and therefore a small nasal airway (midface deficiency), suggesting increased upper airway resistance [35]. A small or narrow upper arch of teeth in the maxilla suggests that the mandible is retrognathic if it is occluding behind the teeth of the maxilla. This causes the tongue to obstruct the oropharyngeal airway and predisposes the child to have OSA [36]. A class II malocclusion is often a common finding. These affected children develop a long craniofacial profile, including retrognathism of the mandible, and midface hypoplasia is often seen. Maxillary crossbite may also be seen. Other more subtle features may include speech difficulties.

The relationship between the upper and lower teeth can indicate either maxillary deficiency or mandibular discrepancy to the cranial base or both and can be diagnosed by the dentist. This can present as malocclusion or ‘bad bite’. If the maxilla is deficient laterally (narrow arch), then the mandible is forced posteriorly for the teeth to fit. This is a class 2 malocclusion. If the maxilla is deficient anterio-posteriorly, then the bottom teeth might either be forced back and tip posteriorly (retroclined) or completely jump past the upper teeth to form a class 3 malocclusion. The teeth can bend forward or back within the arch of bone to try to fit together. Ideally, they should be optimally positioned in the alveolar bone to enable vertical loading during function. Class 1 occlusion is considered normal, however, if the maxilla and mandible are both posteriorly located (closing the airway), then it is class 1 malocclusion (also referred to as class 4 malocclusion by Dr. Kevin Boyd). Commonly, there will be no room for wisdom teeth to erupt, rather, there will be cross bites, rotations, and other forms of crowding because the anatomy (and therefore function) is not optimal. Abnormal craniofacial morphology has been linked to obstructive sleep apnea [37].

Abnormal features in infants as early as six months that shows a long face, retroposition of the mandible, small triangular chin, long uvula clearly behind the tongue, low placed hard palate, overall small upper airway, tonsils present, flaring nostrils when supine and breathing through the nose were all features associated with apparent life threatening events and sleep apnea early in childhood [10].

Abnormal postures of oral circumferential muscles may lead to malocclusion in early childhood. These postures include forward tongue thrust, tongue biting, and low tongue at rest. Maxillofacial structure, oral function, and oral posture are interrelated factors that work together to maintain normal occlusion. Disruption of any of these factors may contribute to malocclusion and impact dentition in children [38,39]. Furthermore, low tongue position can also be seen in OSA patients. Low tongue position is associated with the development of abnormal palate shape and narrowed maxillary dental arch [40,41].

There are studies that have shown that OSA patients also have an extended head posture. This can influence the development of abnormal craniofacial skeleton and dental occlusion. Although children with malocclusion have the same characteristics as patients with OSA, to our knowledge there is no literature that proves the relationship between these two conditions [40,41].

Functional Airway Evaluation Screening Tool (FAIREST-6) is a validated tool used to identify red flags for SDB in children during extra oral and intra oral dental examinations. The following factors had the strongest impact on the Sleep Disturbance Scale for Children (SDSC): mouth-breathing (functional), mentalis strain (extra-oral), tonsillar hypertrophy and ankyloglossia (intraoral soft tissue), dental wear, and narrow palate (intraoral hard tissue). This concise and validated clinical assessment tool may be beneficial in reviewing potential red flags of the craniofacial complex to screen for SDB, aiding in early diagnosis and intervention [42].

Along with the primary care provider, the dentist is also an ideal healthcare provider who can screen for OSA and intervene early since not only can they screen healthy individuals in their dental clinics, but can also assess these craniofacial features that are associated with OSA. Dentists can help collaboratively and proactively in the diagnosis of OSA along with physicians. Clinical features and history can be easily captured in a routine dental admission history to complete the screening of potential sleep apnea and initiate collaboration with sleep medicine specialists.

## 5. The Role of the Dentist

Patients go to their dentist when they are healthy to prevent problems with their teeth and optimize the aesthetics of their smile. This includes evaluating the role of the teeth, muscles, and jaws as they contribute to the size, which is captured in routine dental photography and assessment on admission to a dental clinic. The airway is routinely assessed and followed up as the craniofacial region grows and develops in children once or twice per year when the child appears for regular dental care. Dentists are in the ideal position to capture growth points photographically early on and compare them to previously documented subtleties and help the child get on/stay on track, along with the primary care provider or pediatrician, for optimal health. Dentists are also ideally situated to identify deviations from normal early on, including screening for medical problems as dentists routinely do a medical history on admission and update it at each visit. This is ideal for patients who do not see a medical doctor routinely and goes only as needed [11].

Dentists can recognize and help prevent abnormal development of the jaws and help people keep all their teeth until old age. Since they are regularly involved in oral health, they are also important team players for the early detection of features associated with OSA. Dentists could detect abnormal anatomy and signs and symptoms of abnormal function that is a risk factor for OSA (enlarged tongue, tongue-tie, missing teeth, small and malpositioned jaws) and other signs of the possible cause of sleep disruption (bruxism, temporomandibular joint disorders) [43,44,45].

A policy statement from the American Academy of Dental Sleep Medicine states that dentists play an integral role in reducing the public health burden of undiagnosed and untreated sleep-related breathing disorders [46]. Dentists are encouraged to document abnormal anatomy of the jaws, teeth, and upper airway, screen patients for OSA with questionnaires, and collaborate with physicians for diagnosis and treatment. Physicians are responsible for the diagnosis and prescribing appropriate treatment [7] while also considering the implications of underdeveloped craniofacial anatomy. Collaboration between the sleep medicine physician and the dentists is ideal to address the form and function of the airway, and document and assess the impact on the possible reversal and prevention of obstructive sleep apnea. 

This connection between optimal jaw development, position, and optimal function has been demonstrated in adults clinically in several single case studies and small cohorts by dentists [35,47,48,49,50] based on basic anatomy and physiology [20,51]. 

## 6. Diagnosis of OSA

The diagnosis of obstructive sleep apnea is based on a comprehensive evaluation that includes a history and physical exam as well as investigations to confirm OSA, including an overnight polysomnogram reviewed by a sleep medicine physician. A detailed medical and sleep history, as well as an oral cavity examination is needed. Dentists as well as primary care providers and/or pediatricians play a crucial role in screening for OSA as they often see the patient twice a year for an exam. In general, sleep-trained dentists are familiar and do play an active role, but general dentists may not. Non-sleep-trained dentists may not routinely screen for OSA yet, but it is encouraged by the American Dental Association [31]. The best two questionnaires such as Chervin’s sleep questionnaire (PSQ) and OSAS-18 in pediatrics only have a sensitivity and specificity for detecting OSA of 72–87%. Some dentists have started using such questionnaires in practice in an effort to screen for OSA. Medical questionnaires do not take into account dental findings that may support a diagnosis of OSA. Even the best available questionnaires do not account for features related to the craniofacial profile, such as malocclusion discussed above, asthma, allergies, and/or gastroesophageal reflux. Questionnaires alone are not sufficient to diagnose OSA but can be a good screening tool in an office setting [52].

Overnight polysomnographic studies are still considered the gold standard for diagnosis in addition to a comprehensive sleep assessment as mentioned. Polysomnography thresholds for OSA differ in pediatrics compared to adults. The apnea index for children > 1 or apnea hypopnea index > 1.5 are considered abnormal. The commonly used classification scheme for OSA in children is as follows: Mild AHI 1–5, moderate > 5–10, and severe > 10. These are arbitrary thresholds and have not been linked to end-organ dysfunction. In addition to an overnight polysomnogram, diagnostic imaging, including cone-beam CT can be helpful to evaluate the craniofacial airway in more detail when dental treatment options are considered. The concern about the amount of radiation over a lifetime and risks versus benefits of CBCT should be considered [53]. Drug-induced sleep endoscopy is gaining popularity in children with residual OSA and children with increased risk of persistent OSA despite T&A and helps determine the role of adenotonsillectomy [54].

Gozal et al. proposed several criteria, classified into major and minor, for the diagnosis of OSA in children and to assess the need for treatment. The major ones include an AHI > 2, RDI > 2, Nadir SpO2 < 90%, excessive daytime sleepiness, academic difficulties, hyperactive behavior, hypertension, enuresis, and obesity. Among the minor ones, there are high levels of c-reactive protein (CRP), low density lipoprotein (LDL), fasting insulin, and low levels of high density lipoprotein (HDL), recurrent middle ear otitis, and adenotonsillar grade > 1. The positivity of five major criteria, or three major criteria plus three minor criteria, indicates the need for therapeutic procedures [55]. Their paradigm, although promising, has not been systematically evaluated prospectively.

The most recent guidelines from the American Academy of Pediatrics still endorse a comprehensive evaluation followed by an overnight polysomnogram to establish the diagnosis of OSA. Polysomnography represents the gold standard and involves overnight monitoring of respiratory and sleep-related parameters with direct visualization of the patient during sleep study by the sleep technologist [56]. The cumbersome nature and cost of this test has resulted in innovation to develop more easily accessible ways to diagnose OSA in children. Although pulse oximetry has limited sensitivity and specificity, it has been used to diagnose OSA in children when PSG is not available [57]. The use of the pediatric sleep questionnaire with pulse oximetry has been recommended when polysomnography is not available. However, pulse oximetry can miss OSA in upwards of 40% of children. More recent advancements in wearable technology using cardiopulmonary coupling such as Sleep Image have yielded results similar to that of overnight polysomnography in children, and it is a validated tool approved by the United States Food and Drug Administration (FDA) and Health Canada. Its role in the general clinical setting is not yet established [58,59]. Newer technology is emerging that may allow a clinically acceptable and timely sleep study result for diagnosis and monitoring of treatment. Physicians may be able to diagnose OSA and counsel patients via telemedicine to allow for an easy referral from the dentist for collaborative diagnosis and treatment for the patient.

## 7. Medical Management of OSA

Medical management (non-dental treatments) of OSA in children involves adenotonsillectomy as the first line of treatment. Early systematic reviews suggested that adenotonsillectomy resulted in the normalization of polysomnographic findings in 79% of the children in the Childhood Adenotonsillectomy Trial (CHAT) study [60]. The CHAT study also showed that in children with mild OSA, 46% of the sleep studies normalized in seven months with watchful waiting. However, the study did not show significant improvement in attention or executive function as measured by neuropsychological testing. So, the option to either have the surgery done or perform ‘watchful waiting’ still exists depending on the provider’s preference. Orthodontic treatment was not mentioned in the paper and maxillary development may need to be considered. In this context, the treatment approach for mild OSA is now fraught with debate as to what is considered optimal treatment.

Inflammation is reported to be present in the adenotonsillar tissues and upper airways in children with obstructive sleep apnea [61]. Medical management of mild sleep disordered breathing can include administration of targeted anti-inflammatory therapy. Several studies have used intranasal steroids and leukotriene receptor antagonists to treat mild OSA over the past 15 years [62]. CPAP or continuous positive airway pressure therapy has also been used to treat OSA in children and has been linked to improved sleep quality and neurocognitive outcomes. The compliance rates reported in the literature for PAP therapy for OSA are variable and can range from 30% to 85% [63,64,65]. The long-term impact on the craniofacial skeleton is a concern. Weight loss is recommended for obese children as adenotonsillectomy alone is less likely to result in a complete cure in obese children.

Given the reported sequelae linked to OSA in various physical domains, it stands to reason that early identification and treatment are imperative to prevent morbidity and potentially mortality linked to OSA. OSA should be treated as early as possible to reduce behavioral issues in children [66]. In a large population based study, early life SDB symptoms had strong effects on subsequent behavior in childhood [67]. Potentially mitigating this trajectory with collaboration between medicine and dentistry is promising.

## 8. Dental Treatments

Dental treatments for OSA include many forms of dental devices or oral appliance therapy (OAT). These include growth modifiers of the maxillary region and/or mandibular region, mandibular advancement appliances (M.A.D.), tongue retaining devices, and myofunctional appliances. Some of these devices help make room for teeth using tooth mechanics involving inflammation, while other devices, such as a M.A.D., simply work by holding the lower jaw forward [68]. Characteristic facies of children with OSA often include maxillary constriction, high arched palate, narrow maxillary arch width (with accompanying distalization of the mandible and its soft tissues toward the airway), and maxillary crowding, as well as midfacial hypoplasia, as discussed previously. There are limited studies on the use of dental devices in children, however, novel treatments to prevent braces in dentistry, such as early interceptive treatment to help optimize jaw growth and craniofacial development, are increasing. The premise is to assist the growth of the upper jaw and optimize the position of the lower jaw. The intercuspation of the teeth provides the stimulation of the gnathological biofeedback system [20]. This allows the tongue to have enough space to function optimally and the teeth to come together comfortably into class 1 occlusion. This may reduce accommodation and the need for inflammatory intervention (surgery, pain) ultimately permitting better anatomy for sleep optimization.

### 8.1. A. Rapid MaxillaryExpansion or Rapid Palatal Expansion

Rapid maxillary expansion (RME) or Rapid palatal expansion (RPE) is an orthodontic procedure that uses a fixed or a removable appliance that widens the two halves of the maxilla at the mid-palatal suture line in children with maxillary arch constriction. The expansion screw is progressively adjusted to open the palatal suture. Osteiods develop at the borders of the palate, which results in an increase in the transverse width of the airway. The expected result will increase in oropharyngeal volume and intranasal diameter and decrease nasal resistance, by improving laminar airflow in the nose, which will help in the treatment of OSA. This procedure is used in children when the palatal suture is still open, until around the age of 12 yrs. However, it is accompanied by a loss of anterior-posterior dimension [69]. This would cause the distalization of the mandible moving the tongue back into the airway. Another caveat is that there must be teeth for the expander to anchor to so very young children would not be able to be treated with this. The data on the age of the patient that can be treated using this dental device is limited to children over 4 yrs.

The use of RME in children has shown favorable results, especially in the treatment of residual OSA post T&A for patients with narrowed upper palate [70]. A meta-analysis in 2017 by Guilleminault has shown that RME is effective in substantially reducing the AHI and improving oxygen saturation in children (7.6 ± 2 years old) with OSA and high arch and/or narrow hard palates, especially in the short term (<3 years) follow-up. The improvement was seen more in children with previous adenotonsillectomy or small tonsils than in children with large tonsils [70].

A more recent meta-analysis published in 2021 involving 5- to 13-year-old children showed RME increased the internasal and inter-zygomatic distances and oropharyngeal volume after RME treatment. The effectiveness of RME varies at what age it is done. Although this meta-analysis showed that the quality of the evidence for each outcome in the study was very low, the report of the clinical improvement appears to be favorable. One study included in this meta analysis indicated that in those over 14 years of age, RME would not achieve ideal results due to the degree of established ossification at this age. In adults where the ossification process has occurred in the mid palatal suture, RME can only be achieved by surgically assisted RME [71].

Several long term studies in children have shown that the RME is able to decrease the AHI in children with OSA as well as increase oxygen saturation. One study evaluated RME as a long-term treatment option for OSA and found that there were consistent improvements in polysomnography values after a 12-year follow-up in children with OSA without enlarged tonsils and adenoids [72]. Another study showed long-term follow-up on children for up to 24 months and showed no relapse after treatment of OSA with RME [70]. Adenotonsillectomy, as well as RME, were both done for complete resolution of OSA in one study [73]. Thus, RME may be considered as an alternative to adenotonsillectomy or can be offered when there is residual OSA post T and A in the treatment of OSA in pediatric patients with transverse maxillary deficiency [70].

A study that used RME to treat sleep bruxism was performed on 32 patients between 8–14 years old and showed that it reduced bruxism, but the sleep and respiratory variables remained unchanged [74].

The responsibility for the effectiveness of RME includes not only the provider but also the patient and the parent or caregiver to manage intramural activation of expansion screws, appliance dislodgement, retention of correction after expansion, and complications. After the appliance is applied, the caregiver/parent needs to activate the expansion screw by turning it once or twice a day according to the practitioner’s recommendations. To avoid loss of correction, a retention protocol must be followed. This includes keeping the expander in place until comprehensive orthodontics evaluation is undertaken. Since expansion forces are applied to the teeth at a distance coronal to their rotation center, teeth will tip buccally. The absence of suture release during expansion will lead to buccal crown tipping attached to the expander and minimal to no skeletal expansion, making it prone to relapse of the accomplished expansion. Overall, the majority of the approaches involving RME from tads (screws) to surgery can improve results and do show durability but are also inflammatory in nature [75,76].

Little research has been carried out when it comes to the risk of root fenestrations or dehiscence as the roots of teeth are displaced buccally toward the cortical plate. The bone is less mature and mineralized among young patients; hence, it was speculated that there is a greater risk of expansion, however, less force is needed to separate midline palatal sutures, and children may tolerate such forces better than patients with mature alveolar bone. Clearly, more research is needed to guide decisions about the timing of early intervention [70]. 

### 8.2. B. Complete Airway Repositioning and/or Expansion (CARE)

Another novel treatment option that involves maxillary development is the DNA device by Vivos therapeutics, CARE (Complete Airway Repositioning and/or Expansion) previously called Biomimetic Oral Appliance Therapy (BOAT Therapy) which uses concepts of non-inflammatory body modification by tapping into the biofeedback systems. This treatment will optimize one’s genetic potential [77] and can address midfacial deficiency and narrow palates that can result in tooth crowding and possibly obstructive sleep apnea. CARE can stimulate the body’s natural feedback mechanisms to change form (increase the size of the maxilla) and optimize function (for eating, increased nasal airway, forward posture of the mandible increasing the oral pharyngeal airway for breathing) [78]. The hypothesized mechanism of action is non-inflammatory distraction osteogenesis combined with signal transduction. This enhances the biofeedback mechanisms of the craniomandibular system to allow for de-accommodation of the phenotype and movement toward the genetic potential, which is to have 32 fully erupted teeth in a class 1 occlusion with a healthy upper airway [78]. Evidence of noninflammatory protocols is starting to emerge as beneficial for bone development in the craniofacial region [79].

The DNA device (used in CARE) is FDA-registered as a class 1 device for maxillary expansion and can be used to provide therapy to assist with the development of the maxilla by function in children in a non-inflammatory way. It is worn 12–16 h/day. It has been observed that OSA in children decreases as the maxilla develops to fit the teeth (unpublished data, Heit et al.). This device can also be used in adults to treat OSA [35]. 

Braces or Invisalign are used in phase 2 to straighten the teeth within the alveolar bone and finish the occlusion once maximum medical improvement is achieved (less pain measured by HIT-6 scores and less obstructive sleep apnea measured by AHI). When CARE therapy is implemented, the final physiologic rest position of the jaw can be determined using EMGs, jaw tracking, and sonography. Then, the teeth are mechanically aligned, cusp to fossa to support the healthier jaw position. The hope is that less compensation is required when the hard tissues (all bones and teeth) are fully developed and aligned. Braces and maintenance of the occlusion are required for long-term stability because relapse is very common in orthodontics [80]. The explanation for relapse is that the patient has not attained functional balance in the physiologic rest state. More definitive studies including clinical trials are underway to determine the role of this therapy in treating OSA.

### 8.3. C. Mandibular Advancement Devices (MADs)

Mandibular advancement devices (MAD) or mandibular advancement splints (MAS) can prevent upper airway collapse by means of protruding the mandible forward, hence altering the jaw and tongue position in adults, potentially in older teens, but not in young children. This is currently only approved in adults with mild to moderate OSA. However, with the advent of easier sleep study approaches, dentists can test the effectiveness and support the addition of mandibular advancement device (adding a lower device) in children if it improves their symptoms, the metrics in their sleep study and allows for the developing maxilla and its dentition.

MAD can increase airway volume at the velopharynx. Studies involving MAD mostly defined treatment success as achieving AHI of usually ≤5–10/h or a certain percentage reduction in AHI (usually 50%), and studies have shown a mean AHI reduction of between 30% and 72%. Comparing it to CPAP, results demonstrate that CPAP is more effective than MAD at reducing OSA and achieving complete control of OSAHS (AHI < 5), but it is not more effective when it comes to health outcomes since the higher efficacy of CPAP is offset by greater MAD compliance [81]. Long term side effects of MAD therapy are dental and skeletal changes, which are progressive over time [82]. It stands to reason that combination therapy of CPAP and MAD may also be considered as the CPAP might be more comfortable at lower therapeutic pressures resulting in improved sleep metrics [83].

The MADs hold the lower jaw forward and do not work if they are not in situ [84]. They help to keep the airway open during sleep by using the top jaw as an anchor. However, long term, there are potential craniofacial and bite changes as the body compensates for the design of the constant forces of the device itself that do not address the root cause of the structural problem in the tissues (i.e., a tongue tie or deficient maxilla). Craniofacial features that include retrognathism of the mandible are also associated with OSA. Studies that have looked at increasing the retropharyngeal space or overall airway size with mandibular advancement devices using different modalities have also been studied. The different devices used have included modified monobloc or activated headgear. Amended analysis performed to evaluate functional appliances on upper airway dimensions and growing children with class II malocclusion showed that functional appliances can also increase airway dimensions and may decrease the risk of obstructive sleep apnea. Functional appliances that minimize surgical interventions are preferred as they are non-invasive and can address the size of the upper jaw, thereby affecting the posture of the lower jaw. Maxillary development increases nasal functional space and allows the lower jaw to come forward, opening the upper airway system in two spots—the nasal airway and the oropharyngeal airway. This is thought to improve OSA [81].

## 9. Role of Myofunctional Therapy as an Adjunct to the Dental Plan

Orofacial myofunctional therapy (OMT) is an exercise-based therapy to re-train proper, eating, chewing, swallowing, speaking, and normalizing resting postures that include lip closure, tongue fully seated on the palate, teeth slightly apart, and nasal breathing. It is designed to re-establish the normal oral function of the tongue and orofacial muscles. OMT also works to eliminate maladaptive habits such as thumb sucking, nail-biting, and tongue thrusting that can have negative effects on the craniofacial structure. This, in conjunction with other medical and/or dental therapies, can be an adjunct to treating OSA. Orofacial myofunctional disorder (OMD) involves dysfunction of the tongue, lips, jaw, and other oral structures acquired from critical factors in the growth and development of the orofacial complex from infancy to childhood. Possibly affected functions from abnormal oromotor functioning may include difficulty breastfeeding, airway obstruction, soft tissue restriction, mouth breathing, altered or abnormal oral resting postures, abnormal oral habits, swallowing dysfunction, and altered mastication [85]. Many disregard the importance of the orofacial muscles, however, these muscles are part of the upper airway anatomy and need to be considered. The tongue can play a major role in airway obstruction in OSA. Orofacial myofunctional exercises are designed to improve airway function and balance of the facial muscles that are involved in swallowing, breathing, speaking, and chewing [86]. These isometric and isotonic exercises aim to promote proprioception, range of motion, coordination, and strength of the orofacial muscles to work towards establishing proper eating, chewing, and swallowing which can have a positive effect on craniofacial growth in children. During a normal swallow, the back and base of the tongue connects with the soft palate which aids in keeping patency to the muscles of the soft palate [87]. The aim of myofunctional therapy is to cause the soft palate to elevate, which recruits different upper airway muscles, and muscle training is involved. The exercises are meant to improve muscle fatigue in OSA patients and help improve muscle strength in different pharyngeal segments. One of the ultimate goals of OMT is to allow the tongue to retain the gains from the rapid palatal expansion procedure discussed earlier. Myofunctional therapy can also reduce the volume and fat in the pharyngeal structure and muscles, thus decreasing upper airway collapsibility [88]. Additionally, weight loss has been found to improve OSA through the reduction of adipose tissue in the upper airway–tongue fat–and abdominal fat, as mentioned previously. The decrease in tongue fat aids in the improvement of the apnea hypopnea index, alleviating symptoms of OSA [8].

Myofunctional therapy has been explored as a nonsurgical approach to managing OSA. In a study of 54 children with OSA, myofunctional therapy led to improvements in mean oxygen saturation and the desaturation index in the treatment group [88]. One meta-analysis study showed a decrease in AHI by 50% in adults and 62% in children. Furthermore, polysomnographic studies indicated a 72.4% reduction in snoring and improvement in oropharyngeal muscle tone, suggesting the use of myofunctional therapy as a possible adjunct to OSA treatment [89]. Treatment using these exercises is aimed to reduce mouth breathing and improve the tongue position at rest and during sleep, especially in the context of dental skeletal malocclusion. There have been improvements noted when nasal breathing has been recovered [90,91,92]. The idea behind these interventions is that function alters form and form affects function, and these interventions support a bidirectional model of care. An additional benefit of myofunctional therapy is demonstrated in a study of adults, reporting compliance to CPAP in combination with myofunctional therapy (65%) versus CPAP alone (30%). Thus, myofunctional therapy may provide benefits in promoting quality of life and adherence to CPAP in patients with OSA [88].

## 10. Interdisciplinary Approach

The early screening of pediatric OSA is paramount for timely diagnosis and management. Dentists play a vital part in this preventative role as they can provide another portal for screening for OSA. According to Greenberg and Glick, dentists are confused about the contradictory data in publications and are hesitant to change their practice until more data is available [11]. Recent interdisciplinary collaborations to get appropriate data driven protocols and publications in place will help the conundrum of protocols without data and data needed to make protocols. Screening is best achieved by dentists, primary care providers, and pediatricians involved in regular childcare. For children, it is a timely diagnosis that if not intercepted can affect them for the rest of their lives. Dentists and orthodontists play a critical role in the early identification of craniofacial problems associated with pediatric OSA. They may also play a role in the management of craniofacial abnormalities identified in children that have been diagnosed and treated with OSA post-tonsillectomy. The collaboration of medical and dental professionals is imperative to optimize airway functioning and prevent associated morbidities.

The gap in current dental literature regarding pediatric OSA will improve once these collaborative efforts are realized, documented, and published. Education for medical doctors and dentists will evolve quickly when the licensing bodies are able to legislate data-driven training guidelines for their members in the medical and dental professions. Patients want to be healthier and feel better. Parents want to prevent problems developing in their children while reducing dental costs from complex treatment plans that result from underdeveloped jaws. Often providers caring for the same patient may differ in their understanding of what the other can contribute. It will be a helpful practice if the communication of the patient’s condition from the dental perspective is provided to the medical doctor taking care of the patient. This will result in well-coordinated care of the individual patient as well as overall organization and tracking of multiple patients in a busy dental practice.

Overall, OSA is a very hot topic in both medicine and dentistry, but the uptake of mass training is currently lagging as the majority of the members await a protocol that is data-driven and has significant scientific evidence. It is shown that the difficulties dentists have are related to a lack of up-to-date evidence for treatment, conflicting evidence in the literature, and a lack of clear answers to clinical questions [93]. Dentists and medical doctors need to work together by referring to each other, gathering data together to assess the effectiveness of treatment, and publishing their results together to help dentists become part of the front line in screening for sleep disordered breathing.

## 11. Conclusions

Children suffering from OSA and various forms of SRBD are best managed by a multidisciplinary team consisting of a pediatrician, ENT specialist, a pediatric dentist/orthodontist, a myofunctional therapist, and a sleep medicine physician to manage all the downstream effects of abnormal anatomy and function that have led to the disease. Dentists can help via early detection of these anatomical changes and intercept with treatment to optimize anatomy and function during a child’s growth and development. Properly trained dentists can play a critical collaborative role in OSAS detection, prevention, and management of the dental-skeletal factors, while the medical and surgical aspects are managed by sleep medicine physicians and surgical colleagues, respectively.

Everyone should screen for obstructive sleep apnea and know where their patients can get help.

## Data Availability

Not applicable.
